# Effects of Additives on the Mechanical and Fire Resistance Properties of Pultruded Composites

**DOI:** 10.3390/polym15173581

**Published:** 2023-08-29

**Authors:** Natalia Romanovskaia, Kirill Minchenkov, Sergey Gusev, Olga Klimova-Korsmik, Alexander Safonov

**Affiliations:** 1 Center for Materials Technologies, Skolkovo Institute of Science and Technology, 30/1 Bolshoi Boulevard, 121205 Moscow, Russia; natalia.romanovskaia@skoltech.ru (N.R.);; 2 World-Class Research Center “Advanced Digital Technologies”, State Marine Technical University, 3 Lotsmanskaya Street, 190121 Saint Petersburg, Russia

**Keywords:** glass fibers, pultrusion, fiber reinforced polymers, mechanical properties, flame retardant, fire behavior, ignitability, flammability, combustibility

## Abstract

Under high temperatures, fiber-reinforced polymers are destroyed, releasing heat, smoke, and harmful volatile substances. Therefore, composite structural elements must have sufficient fire resistance to meet the requirements established by building codes and regulations. Fire resistance of composite materials can be improved by using mineral fillers as flame-retardant additives in resin compositions. This article analyzes the effect of fire-retardant additives on mechanical properties and fire behavior of pultruded composite profiles. Five resin mixtures based on vinyl ester epoxy and on brominated vinyl ester epoxy modified with alumina trihydrate and triphenyl phosphate were prepared for pultrusion of strip profiles of 150 mm × 3.5 mm. A series of tests have been conducted to determine mechanical properties (tensile, flexural, compression, and interlaminar shear) and fire behavior (ignitability, flammability, combustibility, toxicity, smoke generation, and flame spread) of composites. It was found that additives impair mechanical properties of materials, as they the take place of reinforcing fibers and reduce the volume fraction of reinforcing fibers. Profiles based on non-brominated vinyl ester epoxy have higher tensile, compressive, and flexural properties than those based on brominated vinyl ester epoxy by 7%, 30%, and 36%, respectively. Profiles based on non-brominated epoxy resin emit less smoke compared to those based on brominated epoxy resin. Brominated epoxy-based profiles have a flue gas temperature which is seven times lower compared to those based on the non-brominated epoxy. Mineral fillers retard the spread of flame over the composite material surface by as much as 4 times and reduce smoke generation by 30%.

## 1. Introduction

Fiber-reinforced polymer (FRP) structures are widely used in civil engineering, architecture, and the automotive industry [1,2,3,4,5,6,7] due to their high corrosion resistance, low thermal conductivity, and high specific strength [8,9,10,11]. One of the most efficient methods of manufacturing FRPs is the pultrusion process [12,13,14,15]. Pultrusion allows manufacture of composite products with a constant cross section, called profiles. In the pultrusion process, the fiber reinforcement pack impregnated with thermoset resin passes through the heated die block where the polymerization process takes place and a profile assumes its shape [16,17]. Pultruded products have excellent mechanical, chemical, and structural characteristics [13,14]. Basically, in pultruded profiles, unidirectional fibers are used as the longitudinal reinforcement and various woven and non-woven materials as the transverse reinforcement. Currently, pultrusion is used to manufacture complex-shape profiles for structural applications, such as strip [18,19,20], rod [21], L-shaped [22,23], U-shaped [24], I-beam [25], and square [26] profiles.

Structural elements used in civil engineering must meet the fire resistance requirements specified in building codes and regulations [27,28]. Fire engineering is concerned primarily with prevention of injuries and loss of life during a fire [29]. As such, it is aimed at preventing ignition, minimizing the spread of fire and smoke, and extinguishing fire before it becomes widespread. Structural elements must have adequate fire resistance to ensure that structural integrity is maintained for a sufficient period of time for people to escape safely and for firefighters to put out the fire [30]. Fire protection combines active (sprinkler systems) and passive solutions (low-flammability materials and fire-resistant coatings and additives) [31]. To establish fire safety requirements for design of buildings, structures, and fire protection systems, construction materials are classified based on their fire hazard potential [32]. Material usage limitations and possible fields of application are specified in design codes [33,34].

Under heating, fiber-reinforced polymers demonstrate a decrease in strength and stiffness [35,36,37,38]. When exposed to the temperature of resin decomposition, composite materials are destroyed, releasing smoke, heat, and harmful volatile substances [39,40,41,42,43,44]. However, aside from this unfavorable condition, fiberglass materials demonstrate low thermal conductivity [45,46]. Thus, they slow down the spread of fire and resist a burn-through and may serve as a tangible barrier against fire and harmful exhausts [47,48]. To improve fire-resistant properties of composite materials, resin mixtures are usually modified with fire-retardant additives [49]. Brominated flame retardants can be used with a wide range of polymers and are widely available on the market. The general mechanism of such fire retardants is the release of hydrogen halide (HBr) upon thermal decomposition of the polymer. Due to this reaction, the flame becomes unstable, goes out, and reduces heat generation. The disadvantage is a large amount of smoke during combustion. Phosphorus-based flame retardants during thermal decomposition form polyphosphoric acids. Further decomposition leads to formation of unsaturated compounds, followed by charring and formation of a carbonaceous layer with a glass-like coating. This layer is resistant to high temperatures and protects the polymer from exposure to oxygen and thermal radiation. Phosphorus-based flame retardants are effective in materials with high oxygen content such as epoxies and polyesters [50]. The disadvantage of phosphorus-based flame retardants is their high cost compared to halogen-based ones. Flame retardants such as mineral fillers consist of inorganic hydroxides and carbonates [51]. When burned, they decompose into non-combustible gases, water, and metal oxides. The release of water or carbon dioxide during combustion reduces the smoke released by the fire due to the dilution of combustible mass [52,53]. The main mineral fillers are aluminum and magnesium hydroxides and magnesium and calcium carbonates [54,55,56]. The efficiency of mineral fillers becomes noticeable at a mass content exceeding 50% [57]. As mineral fillers replace reinforcing fibers and polymer matrix, they can adversely affect mechanical properties of composite materials. The intumescent flame retardants create a heat-insulating coating of charred substances that prevents further heat penetration into burning polymers [58,59]. These flame retardants have various chemical structures containing phosphorus, nitrogen, and carbon–oxygen, as well as expandable graphite, to form carbon foams. Thermal activation of such chemical compounds should occur early in the fire, and intumescent fire retardants tend to be activated in the range of 160–240 °C, which may preclude their use in high-temperature polymers [31]. A few studies can be found in the literature [60,61,62,63,64,65,66] on the fire behavior and mechanical properties of pultruded profiles used in composite structures that should comply to fire safety requirements. As flame retardants impair mechanical properties of composite materials [67], it is necessary to deeply understand the effect of additives on mechanical and flame-retardant properties simultaneously. Improving and optimizing these properties will extend the scope of materials’ applications and improve reliability of composite structures.

This work investigates the effects of flame retardants on fire behavior and mechanical properties of pultruded FRPs. Five resin mixtures were prepared for pultrusion of 150 mm × 3.5 mm glass fiber-reinforced flat profiles. The first resin mixture is based on non-brominated vinyl ester epoxy and the second one on brominated vinyl ester epoxy. The other resin mixtures are the compositions based on brominated vinyl ester epoxy with different mass content of alumina trihydrate and triphenyl phosphate fillers. A series of fire behavior tests were performed and ignitability, flammability, combustibility, toxicity, smoke generation, and flame spread properties were investigated. Based on the test results, the materials were classified according to their fire hazard potential. Mechanical tests were performed to obtain tensile, compression, interlaminar shear, and flexural properties in longitudinal and transverse directions.

## 2. Materials and Methods

### 2.1. Source Materials

**Figure 1:**
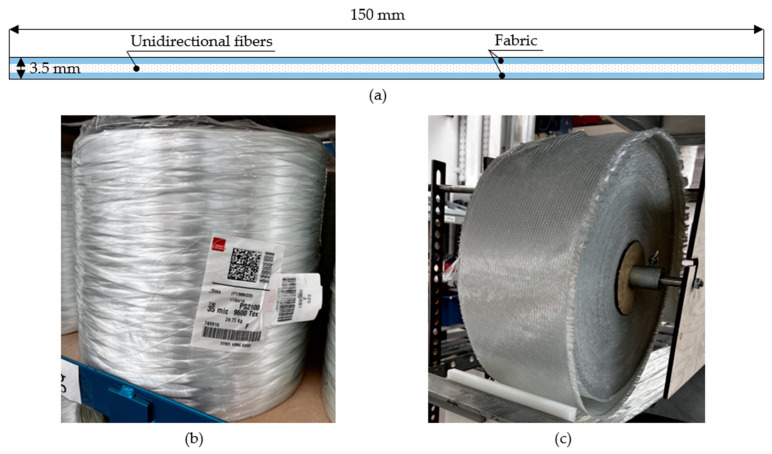
Profile reinforcement: (**a**) scheme, (**b**) glass fiber roving used as longitudinal reinforcement, (**c**) glass fabric used as transverse reinforcement.

The Pulstrand 2100 (Owens Corning, Toledo, OH, USA) 9600 Tex unidirectional glass fiber rovings with filament diameter of 35 microns and two layers of multiaxial non-crimp E-glass fabric with areal density of 900 g/m^2^ LT 0600/S 300/06H 01/125 GUS (Owens Corning, USA) with 0/90 layup were used as reinforcement for pultruded strip profiles with dimensions of 150 mm × 3.5 mm. One fabric layer was placed at the top and at the bottom of the profile for transverse reinforcement. Figure 1 shows the profile reinforcement scheme and used raw materials.

One resin mixture based on Atlac 430 vinyl ester epoxy (DSM Composite Resins AG, Schaffhausen, Switzerland) and four resin mixtures based on Devinil 950 TG brominated vinyl ester epoxy (Dugalak Ltd., Yaroslavl, Russia) were prepared for the study. The following additives were used to obtain optimum resin properties for pultrusion applications: Trigonox C (Akzo Nobel Polymer Chemicals B.V., Botlek Rotterdam, The Netherlands) as an initiator for (co)polymerization of ethylene, styrene, acrylonitrile, acrylates, and methacrylates; Perkadox 16 (Akzo Nobel Polymer Chemicals B.V., Botlek Rotterdam, The Netherlands) as an initiator for suspension polymerization of acrylates and methacrylates; BYK A555 (BYK Additives & Instruments, Wesel, Germany) as a deaerator; and zinc stearate (Baerlocher GmbH, Unterschleißheim, Germany) as a friction-reducing additive [68]. To improve fire behavior, some resins were modified with flame-retardant additives such as alumina trihydrate (Portaflame SG25, Sibelco, Antwerp, Belgium) and triphenyl phosphate (Ambirex TPP). As a wetting and dispersing agent, we used the BYK-W996 additive. The mass content of the components for each resin mixture is shown in Table 1.

The resin components were mixed with an electric mixer (Festool GmbH, Wendlingen, Germany). First, the epoxy resin was added to the mixing container. Next, the main additives were added to the resin as follows: deaerator BYK-A555 (liquid), hardener Perkadox 16 (powder), hardener Trigonox C (liquid), and friction reducing additive zinc stearate (powder). Then, the mixing was started. At the last step, alumina trihydrate (powder) and triphenyl phosphate (powder) flame retardants and dispersing additive BYK-W996 (liquid) were added to the resin, and the components were mixed for 15 min.

### 2.2. Pultrusion Setup

**Figure 2:**
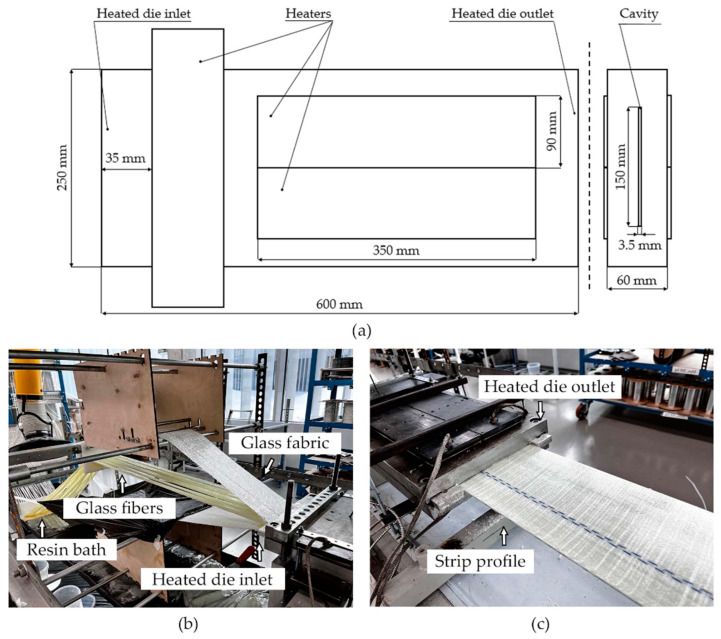
Heated die block for pultrusion of 150 mm × 3.5 mm strip profile: (**a**) schematic representation of the heated die block with heaters, (**b**) fiber impregnation and the die block inlet during pultrusion; (**c**) heated die block outlet and produced profile exiting the die block.

Flat profiles with a cross section of 150 mm × 3.5 mm were produced with the Pultrex Px500-6T pultrusion machine (Pultrex, Lawford, UK). The length of the pultrusion die is 600 mm. Six rectangular heaters 350 mm long and 90 mm wide were used to heat the die block. Two heaters were installed at the top and bottom sides of the die block, aligned with the longitudinal axis of the die. Two heaters were installed at the die block entrance, aligned with the transverse axis of the die. Three thermocouples embedded in the die block were used to control the temperature. The temperature was maintained in the range of 135–155 °C. Figure 2 shows the heated die block used to manufacture the strip profile.

### 2.3. Fire Behavior

Fire behavior is characterized by the following properties: flammability, ignitability, combustibility, flame spread over the surface, smoke generation, and toxicity of combustion products. The material property is described by the hazard group, which is determined based on test results. Based on the combination of its properties, the material is assigned to a fire hazard class. Table 2 shows fire hazard classes of construction materials, based on requirements specified by the Federal Law of Russian Federation № 123-FZ of 22 July 2008 (as amended on 27 December 2018) “Technical Regulation on Fire Safety” [69]. The use of materials of different flammability classes is governed by fire safety requirements. For example, materials rated as KM0 class can be used as floor, ceiling, and wall coverings in living premises, universities, markets, and workshops with a capacity of up to 800 people. Materials rated as KM4 class cannot be used as wall and ceiling coverings. The lower the fire hazard class of materials, the higher the capacity of buildings it can be used in [69].

#### 2.3.1. Combustibility

Material combustibility testing is conducted in accordance with the GOST 30244-94 procedure [70]. The testing setup consists of the combustion chamber, gas burner, specimen holder, and thermocouples. Specimen dimensions are 1000 mm × 150 mm. Specimens are placed vertically in the holder consisting of four rectangular frames located around the perimeter of the ignition source. The burner flame is directed to the lower end of a specimen for 10 min. The test is considered complete after specimens are cooled down to the ambient temperature. The temperature of the flue gases (T), the after-flame time, $t_{af}$ (the duration of specimen smoldering or burning after the flame source is removed), and the loss of mass ($S_{M}$) are measured during the testing. The damaged length ratio ($S_{L}$) is defined as the ratio of the damaged area’s length to the nominal length of the specimen. Materials are divided into four combustibility groups (G1, G2, G3, G4), depending on the determined parameters. Material classification based on combustibility testing results is presented in Table 3.

#### 2.3.2. Ignitability

Material ignitability tests are conducted in accordance with GOST 30402 [71]. The test is used to determine the minimum value of the heat flux density (q) at which a stable ignition and flaming combustion of a material occur. The test setup consists of the radiant heating panel, a gas burner, and a specimen holder. The holder with a specimen is placed inside a truncated radiant heater. The flame of a gas burner is directed onto the surface of a specimen. The gas flow of the burner is set to 19–20 mL/min. Dimensions of test specimens are 150 mm × 165 mm. At the first iteration of the test, the heat flux density of the radiant heater is set to 30 kW/m^2^. The experiment ends when a specimen ignites or after 15 min if a specimen fails to do so. If a specimen is ignited, at the next iteration the heat flux density is reduced. If the material has not ignited within 15 min, the heat flux density is increased at the next iteration. A new specimen is tested at each iteration. The tests are finished when the minimum heat flux sufficient to ignite a specimen is determined. Materials are classified into 3 groups according to their ignitability. Material classification based on ignitability test results is presented in Table 4.

#### 2.3.3. Smoke Generation Index

Smoke generation tests are conducted in accordance with GOST12.1.044-89, Procedure 4.18 [72]. The test is used to determine the smoke generation index based on the optical density of smoke produced by the burning or smoldering material. The test setup consists of a reaction chamber, a controlled flame source, a specimen holder, and a laser system. Specimen dimensions are 40 mm × 40 mm. The smoke generation index is calculated as follows (Equation (1)):

(1) $$D_{m}=\frac{V}{L\cdot m}\ln\left(\frac{T_{0}}{T_{\min}}\right)$$

where $D_{m}$ is the smoke generation index, V—the reaction chamber volume, L—the path length of the light beam in a smoky environment, m—the mass of a specimen before the test, $T_{0}$—the initial value of light transmission, $T_{min}$—the lowest value of the light transmission in a smoke reaction chamber. Based on the calculated index, materials are classified into three groups: low smoke generation (D1), moderate smoke generation (D2), and high smoke generation (D3). Material classification based on smoke generation ability is presented in Table 5.

#### 2.3.4. Toxicity of Combustion Products

Combustion product toxicity tests are conducted in accordance with GOST 12.1.044-89, Procedure 4.20 [72]. The test estimates the amount of lethal poisonous substances released during material burning/smoldering. The test setup consists of the reaction chamber, the radiant heating panel, and the specimen holder. A specimen is placed in the holder and heated to 650 °C by the radiant heating panel. The maximum concentration of carbon dioxide and carbon monoxide released by the smoldering specimen is measured in a closed chamber. Rectangular specimens with dimensions of 40 mm × 40 mm are used in the test. After the testing, the toxicity index (H) depending on the specimen mass, reaction chamber volume, and exposure time is calculated and compared with the average lethal concentration of gases released by the reference material during burning/smoldering. Materials are classified into 4 toxicity groups based on the calculated toxicity index. Material classification based on the combustion product toxicity index is presented in Table 6.

#### 2.3.5. Material Flammability

Flammability tests are conducted in accordance with GOST 12.1.044-89, Procedure 4.3 [72]. The test setup consists of a reaction chamber, tooling to move and fix the specimen, and a gas burner with inner diameter of 7 mm. The reaction chamber is equipped with thermocouples located at a distance of 15 mm from the specimen holder. Before testing, the gas flow rate of the burner is set so that the flue gas temperature does not exceed 200 °C for 3 min. The test begins with introducing the specimen into the reaction chamber so that the flame from the gas burner falls onto the surface of the specimen. Time and temperature measurements start when the specimen is placed inside the reaction chamber. The test ends when the temperature inside the chamber exceeds 260 °C. Should the chamber temperature fail to reach the temperature of 260 °C, the test ends after 300 s. Rectangular specimens with dimensions of 150 mm × 600 mm are used in the test. The temperature change during the test is calculated as follows (Equation (2)):

(2) $$∆t_{\max}=t_{\max}-t_{0}$$

where $∆t_{max}$—the temperature change during the test, $t_{max}$—the maximum temperature of gaseous combustion products of the material tested (reaction chamber temperature), $t_{0}$—the initial temperature of the reaction chamber, equal to 200 °C. After the test, the mass loss (%) of the specimen is calculated as follows (Equation (3)):

(3) $$∆m=\frac{m_{H}-m_{K}}{m_{H}}\times 100\%$$

where ∆m—loss of mass by the specimen, $m_{H}$—specimen mass before testing, $m_{K}$—specimen mass after testing. Based on the calculated temperature change (∆$t_{max}$) and the loss of mass (∆m), a material is classified as non-flammable or flammable. The “flammable” group is further divided into subgroups (poorly flammable, moderately flammable, highly flammable) depending on the heating time required (test time) to reach the highest temperature during the test. The classification of materials is presented in Table 7.

#### 2.3.6. Flame Spread Index

The flame spread index is determined in accordance with GOST 12.1.044-89, Procedure 4.19 [72]. The test setup consists of the pilot burner, the radiant heating panel, the specimen holder, and exhaust hood with a thermocouple. Specimen dimensions are 320 mm × 140 mm. Additional marks (0–9) are drawn on the surface of the specimen at intervals of 30 mm to denote 10 sections. The fixing frame makes it possible to place a specimen at an angle of 30° to the heating panel. Before testing, the heat flow density of the radiant heating panel is set to 32 ± 3 kW/m^2^ when measured at a distance of 70 mm from the closest edge of a specimen. Pilot burner is located at a distance of 8 ± 1 mm from the specimen edge. Pilot burner flame length is set to 11 ± 2 mm. The test starts with placing the fixing frame with a specimen under the pilot burner flame and ends when the flame stops propagating over the surface of the specimen. The flame spread index is calculated as follows (Equation (4)):

(4) $$I={\left[\frac{0.0115\beta }{\tau_{0}}\left(t_{max}-t_{0}\right)\left(\tau_{max}-\tau_{0}\right)\left(1+0.2l\right)\overset{n}{\underset{i=1}{\sum }}\frac{1}{\tau_{i}}\right]}^{0.5}$$

where I—the flame spread index, β—the thermal coefficient of testing equipment, characterizing the amount of heat supplied to the surface of a specimen during the time required to increase the flue gas temperature by 1 °C (in our experiment, β was calculated before the test and constitutes 46 W/°C), $\tau_{0}$—the time the flame front passes the zero mark, $\tau_{1}$—the time the flame front passes the i^th^ section of the specimen surface, l—the length of the flame front path, $\tau_{max}$—the time of reaching the maximum flue gas temperature, $t_{max}$—the highest flue gas temperature, $t_{0}$—the flue gas temperature measured inside the exhaust hood during pilot burner operation without the specimen. Materials are classified according to the value of the flame spread index as follows: non-spreading flame over the surface, slowly spreading, and rapidly spreading. Table 8 shows classification of materials based on the flame spread index.

### 2.4. Material Characterization

Smoke-generating ability, flame spread index, toxicity of combustion products, and flammability were determined in accordance with GOST 12.1.044 procedures [72]. Combustibility tests were conducted in accordance with GOST 30244-94 Method II [70], and ignitability tests were conducted in accordance with the GOST 30402 procedure [71] in the Laboratory of Fire Prevention and New Materials (Tver Institute of Railway Car Building, Tver, Russia).

Mechanical properties of tested profiles were determined with the Instron 5969 testing machine (Instron, Norwood, MA, USA). Compression, tension, flexure, and interlaminar shear tests were performed in accordance with ASTM D6641/D6641M-16 [73], ISO 527-5 [74], ASTM D790-15e2 [75], and ASTM D2344/D2344M-16 [76] procedures, respectively. Series of 10 specimens per profile were tested for each testing procedure.

Specimens for mechanical property and fire behavior tests were machined with the milling machine. Specimens were machined at 10,000 rpm and a feed of 1500 mm/min, using a face mill of 3.175 mm in diameter.

The peak exothermic temperature of resin compositions was determined in accordance with the ASTM D2471 [77] procedure at a bath temperature of 65 °C. The Geltex analyzer (Pultrex, Lawford, UK) was used to analyze reactivity of resins.

## 3. Results

### 3.1. Resin Reactivity

**Figure 3:**
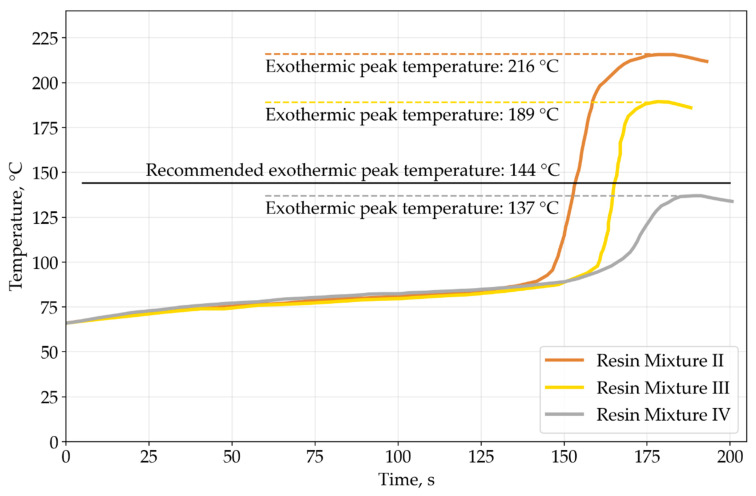
Exothermic peak temperature graph.

As Atlac 430 vinyl ester epoxy resin was already tested in previous pultrusion studies [22], and the pultrusion process is stable, peak exothermic temperature tests were conducted only for resin mixtures II, III, and IV based on Devinil 950 TG brominated vinyl ester epoxy resin. Exothermic peaks at 216 °C, 189 °C, and 137 °C correspond to mixtures II, III, and IV, respectively. Figure 3 shows temperature measurements during the tests. Flame retardants present in the resin mixtures reduce the exothermic peak temperatures. The exothermic peak temperature decreases with an increase in mass fraction of flame retardants. According to the Devinil resin datasheet, the highest mechanical properties of polymerized resin can be achieved at the exothermic peak temperature of 144 °C. The closest value is observed in the case of the resin mixture IV.

### 3.2. Manufactured Materials

**Figure 4:**
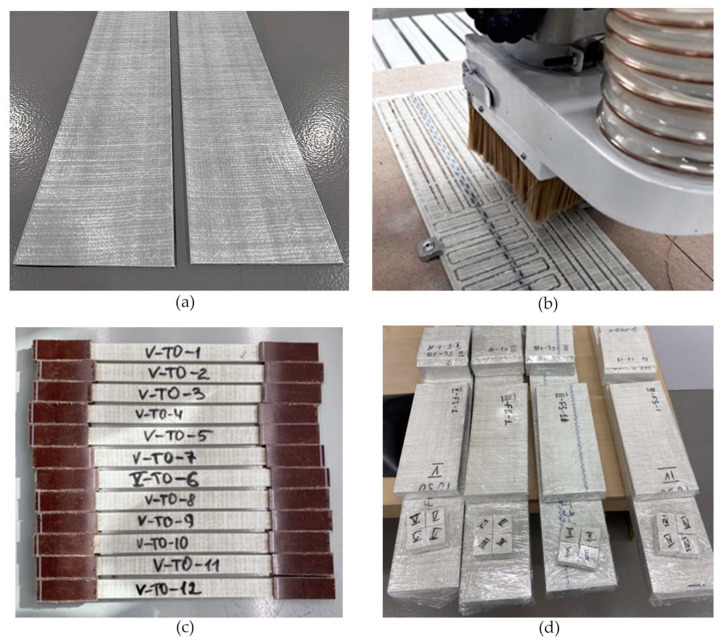
Pultruded profiles and machined specimens: (**a**) pultruded strip profiles, (**b**) cutting of specimens, (**c**) tensile test specimens with tabs, (**d**) specimens for fire behavior testing.

Five batches of profiles were produced at a pulling speed of 0.5 m/min. Profiles are marked according to the resin mixture number—PRM-I, PRM-II, PRM-III, PRM-IV, and PRM-V. The PRM-I profile is based on non-brominated vinyl ester epoxy resin. PRM-II is based on brominated vinyl ester epoxy resin. PRM-III, PRM-IV, and PRM-V are brominated vinyl ester epoxy-based profiles with mineral fillers. In total, 31 m of strip profiles were produced. As fire retardants occupy a certain volume of a profile, the use of additives reduces the volume fraction of reinforcing fibers. Table 9 shows the volume composition of pultruded profiles. Figure 4 shows manufactured profiles and specimens prepared for testing.

### 3.3. Mechanical Properties

**Figure 5:**
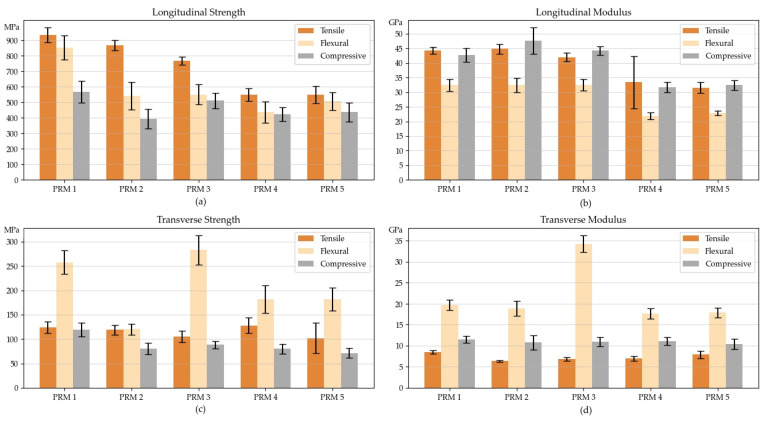
Mechanical properties of pultruded profiles: (**a**) tensile, flexural, and compression strength in longitudinal direction; (**b**) tensile, flexural, and compression modulus in longitudinal direction; (**c**) tensile, flexural, and compression strength in transverse direction; (**d**) tensile, flexural, and compression modulus in transverse direction.

Table 10 shows results of mechanical testing in longitudinal and transverse orientations. Bar charts of mechanical properties are shown in Figure 5. As we can see in Figure 5a, the profiles based on resins modified with additives have lower tensile, flexural, and compression strength in the longitudinal direction compared to those based on unmodified resins. The tensile strength of the PRM-I profile constitutes 934 MPa, which exceeds the strength of PRM-II, PRM-III, PRM-IV, and PRM-V profiles by 7%, 17%, 40%, and 41%, respectively. The flexural strength of the PRM-I profile exceeds that of the other profiles by 36–48% and constitutes 851 MPa. Also, the PRM-I profile has the highest compression strength (568 MPa) exceeding that of the other profiles by 10–30%. Figure 5b shows tensile, flexural, and compression moduli in the longitudinal direction. Tensile modulus differs slightly between PRM-I, PRM-II, and PRM-III profiles and between PRM-IV and PRM-V profiles. The PRM-V profile has the lowest tensile modulus equal to 31.6 GPa. Flexural and compressive moduli differ slightly between PRM-I, PRM-II, and PRM-III profiles, with the lowest values of 31.4 GPa for flexural and 42.7 GPa for compressive moduli. The similar trend can be observed for PRM-IV and PRM-V profiles, with the lowest values of 21.8 GPa and 31.7 GPa for flexural and compressive moduli, respectively. According to Figure 5c, the flexural strength of the PRM-III profile in the transverse direction exceeds that of PRM-I, PRM-II, PRM-IV, and PRM-V profiles by 9%, 57%, 35%, and 35%, respectively, and corresponds to 283 MPa. Only minor differences in tensile strength can be observed. The PRM-I profile has higher compressive strength exceeding that of the other profiles by 8–16%. Figure 5d shows that the PRM-III profile has the highest flexural modulus in the transverse direction as compared to other profiles, while insignificant differences can be observed between compression and tensile moduli. The decrease in mechanical properties can be attributed mostly to a reduced volume fraction of reinforcing fibers, which are replaced by flame-retardant additives.

Theoretical calculations of the tensile modulus were carried out according to the method proposed by Evans [78]. Fabric fiber efficiency β and glass elasticity modulus were taken as 0.7 and 76 GPa [79] in the calculations. As can be seen in Table 10, the experimental moduli of PRM-I, PRM-II, and PRM-III profiles are in good agreement with predicted ones. Experimental values for PRM-IV and PRM-V profiles are higher than predicted ones by 7% and 10%, respectively. Taking the standard deviation into account, the difference between experimental and theoretical data can be considered negligible.

The results of ILSS tests correlate well with results of resin reactivity analysis. Thus, the PRM-IV profile with the highest ILSS values has the exothermic peak temperature very close to the recommended peak temperature. At the same time, the highest exothermic peak temperature exceeding the desired temperature by 72 °C was observed in the case of the PRM-II profile that has the lowest ILSS value.

### 3.4. Fire Behavior Properties

Table 11 shows combustibility test results. The PRM-I profile has a flue gas temperature of 427 °C, which is more than 7 times higher than that of the flue gases released by other materials. The PRM-I specimens were completely damaged by fire (the damaged length ratio is 100%), while the damaged length ratio for other materials constitutes only 30%. The PRM-I specimens lost 13% of their mass during the test, exceeding the loss of mass by other profiles by as much as 3 times. The damaged length ratio and the loss of mass are in a linear relationship. Only slight differences could be observed between flammability properties of specimens based on RM-II–RM-V matrices. All tested materials do not burn unassisted. According to the test results, the profile based on the RM-I matrix falls into G3 combustibility group, and the other profiles (PRM-II–PRM-V) into the G1 group.

Ignitability test results are shown in Table 12. The PRM-IV profile ignited under radiant heating at the highest heat flux density of 30 kW/m^2^. The composite profiles based on the RM-V matrix ignited at a heat flux density of 25 kW/m^2^. Other materials ignited at a heat flux density of 20 kW/m^2^. Thus, all tested materials fall into B2 ignitability group.

Table 13 shows results of smoke generation tests. The highest value of the smoke generation index constituting 325 m^2^/kg was obtained for the profile based on brominated vinyl ester epoxy resin without flame-retardant additives (PRM-II). The lowest value of 226 m^2^/kg was obtained for the glass fiber/vinyl ester epoxy resin profile without flame-retardant additives (PRM-I). The presence of the alumina trihydrate additive in the PRM-III and PRM-IV profiles reduced smoke generation. The increase in the mass fraction of alumina in resin composition reduces the smoke generation index. The PRM-V profile containing both alumina trihydrate and triphenyl phosphate has higher smoke generation compared to the PRM-IV profile with the same amount of alumina trihydrate and no triphenyl phosphate. All tested materials fall into the D2 group of smoke generation ability.

Results of combustion product toxicity tests are shown in Table 14. Experiments were conducted with an exposition time of 30 min. Pultruded profiles fall into the T2 toxicity group of moderately hazardous materials. The index values vary slightly between materials and are closer to the middle of the T2 group range of values, which is 40–120.

Table 15 shows the results of the flame spread test. The lowest values of the flame spread index were obtained for PRM-IV and PRM-V profiles. The high content of alumina trihydrate reduces flame spread over the profile surface. The presence of triphenyl phosphate in the PRM-V profile increases the value of the flame spread index compared to the PRM-IV profile. Tested profiles fall into the slowly spreading group. The range of flame spread index values for this group is 0–20. Pultrusion profiles based on the first three resin mixtures (RM-I, RM-II, RM-III) are in the middle of this range. Profiles based on the last two resin mixtures (RM-IV and RM-V) with a high content of alumina trihydrate are close to the lower boundary of this range (I = 0).

Flammability test results are shown in Table 16. Tests were conducted at an initial chamber temperature of 200 °C. The PRM-I profile has the highest temperature of flue gas of 395 °C and falls into the flammable group, moderate-flammability subgroup. For the PRM-III profile, the flue gas temperature is 268 °C, constituting a temperature difference of 68 °C (the limit value is 60 °C). Therefore, the PRM-III profile falls into the flammable group, moderate-flammability subgroup. The other profiles (PRM-II, PRM-IV, PRM-V) fall into the non-flammable group. The loss of mass during testing for all profiles varies insignificantly and falls in the range of 18.1–20.9%.

Classification of all materials based on fire behavior test results is shown in Table 17. According to smoke generation tests (GOST 12.1.044, Procedure 4.18), all pultruded profiles can be classified as D2 group materials. Combustion product toxicity tests (GOST 12.1.044, Procedure 4.20) show that all materials can be rated as T2 group materials. According to the ignitability tests (GOST 30402-96), all tested materials fall into the B2 group of materials. Results of flame spread tests (GOST 12.1.044, Procedure 4.19) show that all profiles have a slowly spreading flame over their surface. Materials based on resin mixtures RM-I and RM-III can be classified as flammable, moderately flammable materials, while materials based on resin mixtures RM-II, RM-IV, and RM-V are non-flammable materials. According to the results of combustibility tests (GOST 30244-94), PRM-I profiles can be rated as G3 group materials and other profiles as G1 materials. The material based on the resin mixture RM-I can be rated as KM4 hazard class, while other materials as KM2 hazard class. According to of the Federal Law of Russian Federation № 123-FZ of 22 July 2008 (as amended on 27 December 2018) “Technical Regulations on Fire Safety” [69], KM4 class materials can be used as flooring in hotels, hostels, restaurants, and educational institutions with a people capacity of not more than 50. Materials of the KM2 class can be used for flooring in buildings with a people capacity of up to 800. KM4 class materials cannot be used in the buildings listed above as ceilings and walls, while KM2 class materials can be used in buildings with capacity of up to 300 people. The materials based on the RM-I resin mixture present a high fire hazard and their application in building structures is very limited compared to other tested materials.

The highest combustion temperature during flammability and combustibility tests was measured for PRM-I profiles based on bisphenol A vinyl ester epoxy resin. The other tested profiles are based on the brominated vinyl ester epoxy resin and have low flue gas temperature. Brominated resins contain tetrabromobisphenol-A and have flame-retardant properties and higher charring ability [80,81]. According to smoke generation tests, the presence of alumina trihydrate flame retardant reduces smoke generation during combustion and slows down the spread of flame. An increase in the alumina trihydrate mass fraction in the resin mixture reduces the emission of gases and results in reduced values of the smoke generation index.

## 4. Conclusions

This study investigated the effect of flame retardants on mechanical properties and fire behavior of pultruded composite profiles. Five batches of thermoset strip profiles with dimensions of 150 mm × 3.5 mm based on five resin mixtures were produced. Mixtures were based on vinyl ester epoxy resin and on brominated vinyl ester epoxy resin with different content of mineral fillers. Mechanical test results show that the tensile, compressive, and flexural strength of profiles based on vinyl ester epoxy is higher than that of profiles based on brominated vinyl ester epoxy by 7%, 30%, and 36% respectively. However, according to flammability tests, the temperature of the flue gas released by profiles based on brominated epoxy is seven times lower than that of flue gas released by non-brominated epoxy profiles. Non-brominated epoxy-based profiles emit less smoke than those based on brominated epoxy. Profiles filled with alumina trihydrate and triphenyl phosphate had a four times lower flame spread index. The combustion product toxicity index differs slightly between profiles based on non-brominated and brominated epoxy and profiles based on brominated epoxy with alumina trihydrate and triphenyl phosphate. All tested materials are moderately toxic. Fire behavior tests per GOST 30244-94 show that glass fiber-reinforced profiles based on brominated epoxy fall into combustibility Group 1 and are more fire-resistant than non-brominated epoxy-based profiles falling into combustibility group 3. According to GOST 12.1.044 and GOST 30402-96, all tested materials fall into the B2 flammability group, D2 smoke generation group, and T2 toxicity group, and can be classified as slowly spreading the flame over the surface.

## Figures and Tables

**Table 1:** Resin mixture compositions. All values are given in kg.

Resin Mixture	Components								
	Atlac 430	Devinil 950 TG	Trigonox C	Percadox 16	BYK-A555	Zinc Stearate	Alumina Trihydrate	BYK-W996	Triphenyl Phosphate
I	23.39 (93) *	-	0.36 (1.4)	1.22 (4.9)	0.07 (0.3)	0.09 (0.4)	-	-	-
II	-	23.39 (93)	0.36 (1.4)	1.22 (4.9)	0.07 (0.3)	0.09 (0.4)	-	-	-
III	-	23.57 (72)	0.36 (1)	1.24 (4)	0.07 (0.2)	0.09 (0.3)	7.07 (22)	0.14 (0.4)	-
IV	-	23.81 (48)	0.36 (0.1)	1.24 (3)	0.07 (0.01)	0.10 (0.02)	23.81 (48)	0.48 (1)	-
V	-	23.81 (46)	0.36 (0.1)	1.24 (2)	0.07(0.01)	0.10 (0.01	23.81 (46)	0.48 (1)	2.38 (5)
Additive effect	Resin	Resin	Initiator	Initiator	Deaerator	Friction reducer	Flame retardant	Wetting, dispersing	Flame retardant

* Approximate mass fraction (%) is indicated in brackets.

**Table 2:** Fire hazard classes of building materials according to [69].

Fire Hazard Properties of Construction Materials	Fire Hazard Class of Construction Materials, with Corresponding Group Rating					
	KM0	KM1	KM2	KM3	KM4	KM5
Combustibility	NG	G1	G1	G2	G3	G4
Ignitability	-	B1	B2	B2	B2	B3
Smoke generation	-	D2	D2	D3	D3	D3
Toxicity	-	Т2	Т2	Т2	Т3	Т4
Flame spread	-	RP1	RP1	RP2	RP2	RP4

**Table 3:** Material classification based on combustibility test results according to [69].

Material Combustibility Group	Flue Gas Temperature, °C	Damaged Length Ratio, %	Mass Loss, %	After-Flame Time, s
G1	*T* ≤ 135	$S_{L}$ ≤ 65	$S_{M}$ ≤ 20	$t_{af}$ = 0
G2	*T* ≤ 235	$S_{L}$ ≤ 85	$S_{M}$ ≤ 50	$t_{af}$ ≤ 30
G3	*T* ≤ 450	$S_{L}$ > 85	$S_{M}$ ≤ 50	$t_{af}$ ≤ 300
G4	*T* > 450	$S_{L}$ > 85	$S_{M}$ > 50	$t_{af}$ > 300

**Table 4:** Material classification based on ignitability test results according to [69].

Group	Heat Flux Density, kW/m^2^
B1	q ≥ 35
B2	20 < *q* < 35
B3	q < 20

**Table 5:** Material classification based on smoke generation ability according to [69].

Group	Smoke Generation Index, m^2^/kg
D1	$D_{m}$ < 50
D2	$50\;<\;D_{m}$ < 500
D3	$D_{m}$ > 500

**Table 6:** Material classification based on toxicity index according to [69].

Toxicity Groups	Toxicity Index Depending on Exposure Time			
	5 min	15 min	30 min	60 min
T1 (Low hazard)	H > 210	H > 150	H > 120	H > 90
T2 (Moderate hazard)	70 < H ≤ 210	50 < H ≤ 150	40 < H ≤ 120	30 < H ≤ 90
T3 (High hazard)	25 < H ≤ 70	17 < H ≤ 50	13 < H ≤ 40	10 < H ≤ 30
T4 (Extreme hazard)	H < 25	H < 17	H < 13	H < 10

**Table 7:** Material classification based on flammability test results according to [72].

Group	Subgroup	Temperature Change (∆t_max_), °C	Mass Loss (∆m), %	Time (τ), min
Non-flammable	-	$∆t_{max}$ < 60	∆m < 60	-
Flammable	Poorly flammable	$∆t_{max}$ ≥ 60	∆m ≥ 60	τ > 4
	Moderately flammable			0.5 ≤ τ ≤ 4
	Highly flammable			τ ≤ 0.5

**Table 8:** Material classification based on the flame spread index [69].

Group	Flame Spread Index
Non-spreading	I = 0
Slowly spreading	0 < I ≤ 20
Rapidly spreading	I > 20

**Table 9:** Volume composition of pultruded profiles.

	PRM-I	PRM-II	PRM-III	PRM-IV	PRM-V
Matrix	0.37	0.37	0.36	0.37	0.37
Additives	-	-	0.05	0.17	0.20
Reinforcement	0.63	0.63	0.59	0.46	0.43
Number of rovings	62	62	56	37	32

**Table 10:** Mechanical properties of pultruded profiles.

Property		PRM-I	PRM-II	PRM-III	PRM-IV	PRM-V
Longitudinal	Tensile strength (MPa)	934 ± 47	868 ± 34	768 ± 26	550 ± 41	550 ± 55
	Tensile modulus (GPa)	44.3 ± 1.2	44.8 ± 1.7	42.0 ± 1.5	33.4 ± 9.0	31.6 ± 1.9
	Tensile modulus theoretical (GPa)	44.5	44.5	41.3	30.9	28.2
	Compressive strength (MPa)	568 ± 70	394 ± 62	510 ± 49	423 ± 43	436 ± 61
	Compressive modulus (GPa)	42.7 ± 2.3	47.6 ± 4.5	44.2 ± 1.4	31.7 ± 1.8	32.4 ± 1.6
	Flexural strength (MPa)	851 ± 78	540 ± 89	550 ± 64	436 ± 67	507 ± 57
	Flexural modulus (GPa)	32.4 ± 2.1	32.4 ± 2.4	32.5 ± 1.9	21.8 ± 1.2	22.8 ± 0.8
	ILSS (MPa)	33.1 ± 2.9	16.0 ± 1.4	23.5 ± 1.5	30.6 ± 3.0	26.8 ± 1.3
Transversal	Tensile strength (MPa)	124 ± 12	119 ± 10	105 ± 12	128 ± 16	102 ± 31
	Tensile modulus (GPa)	8.5 ± 0.4	6.4 ± 0.2	6.9 ± 0.4	7.0 ± 0.6	7.9 ± 0.9
	Compressive strength (MPa)	119 ± 14	80 ± 12	88 ± 8	80 ± 10	71 ± 10
	Compressive modulus (GPa)	11.5 ± 0.8	10.8 ± 1.7	11.0 ± 1.1	11.1 ± 0.9	10.4 ± 1.2
	Flexural strength (MPa)	258 ± 24	120 ± 11	283 ± 30	182 ± 28	182 ± 24
	Flexural modulus (GPa)	19.7 ± 1.2	18.9 ± 1.8	34.3 ± 2.0	17.7 ± 1.2	17.9 ± 1.2
	ILSS (MPa)	13.0 ± 2.4	14.8 ± 4.9	27.0 ± 9.2	12 ± 2.4	11.3 ± 2.6
Fiber volume fraction		0.63	0.63	0.59	0.46	0.43

**Table 11:** Combustibility testing results.

Material	Flue Gas Temperature, °C	Damaged Length Ratio, %	Mass Loss, %	After-Flame Time, s	Combustibility Group
PRM-I	T = 427 (G3)	SL = 100 (G3)	SM = 13 (G1)	$t_{af}$ = 0 (G1)	G3
PRM-II	T = 59 (G1)	SL = 32 (G1)	SM = 4 (G1)	$t_{af}$ = 0 (G1)	G1
PRM-III	T ≤ 59 (G1)	SL > 29 (G1)	SM = 4 (G1)	$t_{af}$ ≤ 0 (G1)	G1
PRM-IV	T > 57 (G1)	SL > 28 (G1)	SM = 4 (G1)	$t_{af}$ > 0 (G1)	G1
PRM-V	T > 58 (G1)	SL > 25 (G1)	SM = 3 (G1)	$t_{af}$ > 0 (G1)	G1

Three specimens per profile were used for testing.

**Table 12:** Ignitability test results.

Material	Heat Flux Density, kW/m^2^	Flammability Group
PRM-I	q = 20 (B2)	B2
PRM-II	q = 20 (B2)	B2
PRM-III	q = 20 (B2)	B2
PRM-IV	q = 30 (B2)	B2
PRM-V	q =25 (B2)	B2

Minimum of 7 specimens per profile were used for testing.

**Table 13:** Smoke generation test results.

Material	Smoke Generation Index, m^2^/kg	Group
PRM-I	D_m_ = 226	D2
PRM-II	D_m_ = 340	D2
PRM-III	D_m_ = 325	D2
PRM-IV	D_m_ = 241	D2
PRM-V	D_m_ = 281	D2

Three specimens per each profile were tested.

**Table 14:** Toxicity test results.

Material	Toxicity Index	Toxicity Groups
PRM-I	H = 65	T2
PRM-II	H = 72	T2
PRM-III	H = 67	T2
PRM-IV	H = 70	T2
PRM-V	H = 71	T2

Experiments were conducted with exposition time 30 min. Three specimens used for each testing.

**Table 15:** Flame spread test results.

Material	Flame Spread Index	Group
PRM-I	I = 7.2	Slowly spreading
PRM-II	I = 8.8	Slowly spreading
PRM-III	I = 7.9	Slowly spreading
PRM-IV	I = 1.9	Slowly spreading
PRM-V	I = 2.7	Slowly spreading

Series of 5 specimens were used for each test.

**Table 16:** Flammability test results.

**Material**	$\mathrm{Temperature}\;\mathrm{Change}\;(∆t_{max}$), °C	Mass Loss (∆m), %	Time (τ), s	Group, Subgroup
PRM-I	$∆t_{max}$ = 195	∆m = 19.5	τ = 133	Flammable, moderately flammable
PRM-II	$∆t_{max}$ = 47	∆m = 19.1	τ = 300	Non-flammable
PRM-III	$∆t_{max}$ = 68	∆m = 18.1	τ = 107	Flammable, moderately flammable
PRM-IV	$∆t_{max}$ = 57	∆m = 18.5	τ = 300	Non-flammable
PRM-V	$∆t_{max}$ = 58	∆m = 20.9	τ = 300	Non-flammable

Initial chamber temperature is 200 °C. Series of 3 specimens were used for each test.

**Table 17:** Fire behavior properties.

Property	PRM-I	PRM-II	PRM-III	PRM-IV	PRM-V
Combustibility	G3	G1	G1	G1	G1
Ignitability	B2	B2	B2	B2	B2
Smoke generation	D2	D2	D2	D2	D2
Toxicity	T2	T2	T2	T2	T2
Flame spread	Slowly spreading	Slowly spreading	Slowly spreading	Slowly spreading	Slowly spreading
Flammability	Flammable, moderately flammable	Non-flammable	Flammable, moderately flammable	Non-flammable	Non-flammable
Hazard class	KM4	KM2	KM2	KM2	KM2

## Data Availability

The data presented in this study are available on request from the corresponding author.
